# Multi-focal Stimulation of the Cortico-cerebellar Loop During the Acquisition of a Novel Hand Motor Skill in Chronic Stroke Survivors

**DOI:** 10.1007/s12311-023-01526-4

**Published:** 2023-02-18

**Authors:** M.J. Wessel, L.R. Draaisma, M. Durand-Ruel, P. Maceira-Elvira, M. Moyne, J.-L. Turlan, A. Mühl, L. Chauvigné, P.J. Koch, T. Morishita, A.G. Guggisberg, F.C. Hummel

**Affiliations:** 1 Defitech Chair of Clinical Neuroengineering, Neuro-X Institute (INX) and Brain Mind Institute (BMI), École Polytechnique Fédérale de Lausanne (EPFL), 9 Chemin des Mines, 1202 Geneva, Switzerland; 2 Defitech Chair of Clinical Neuroengineering, Neuro-X Institute (INX) and Brain Mind Institute (BMI), Clinique Romande de Réadaptation, École Polytechnique Fédérale de Lausanne (EPFL Valais), Av. Grand-Champsec 90, 1951 Sion, Switzerland; 3 University Hospital Würzburg (UKW), Department of Neurology, Josef-Schneider-Str. 11, 97080 Würzburg, Germany; 4 Department of Clinical Neurosciences, Geneva University Hospital (HUG), Geneva, Switzerland; 5 Clinique Romande de Réadaptation (CRR Suva), Sion, Switzerland; 6 Universitäre Neurorehabilitation, Universitätsklinik für Neurologie, Inselspital, University Hospital of Berne, Berne, Switzerland

**Keywords:** Stroke, Motor learning, tDCS, Cerebellum, M1, Multifocal stimulation

## Abstract

Impairment of hand motor function is a frequent consequence after a stroke and strongly determines the ability to regain a self-determined life. An influential research strategy for improving motor deficits is the combined application of behavioral training and non-invasive brain stimulation of the motor cortex (M1). However, a convincing clinical translation of the present stimulation strategies has not been achieved yet. One alternative and innovative approach is to target the functionally relevant brain network-based architecture, e.g., the dynamic interactions within the cortico-cerebellar system during learning. Here, we tested a sequential multifocal stimulation strategy targeting the cortico-cerebellar loop. Anodal transcranial direct current stimulation (tDCS) was applied simultaneously to a hand-based motor training in *N* = 11 chronic stroke survivors during four training sessions on two consecutive days. The tested conditions were: sequential multifocal (M1-cerebellum (CB)-M1-CB) vs. monofocal control stimulation (M1-sham-M1-sham). Additionally, skill retention was assessed 1 and 10 days after the training phase. Paired-pulse transcranial magnetic stimulation data were recorded to characterize stimulation response determining features. The application of CB-tDCS boosted motor behavior in the early training phase in comparison to the control condition. No faciliatory effects on the late training phase or skill retention were detected. Stimulation response variability was related to the magnitude of baseline motor ability and short intracortical inhibition (SICI). The present findings suggest a learning phase-specific role of the cerebellar cortex during the acquisition of a motor skill in stroke and that personalized stimulation strategies encompassing several nodes of the underlying brain network should be considered.

**Supplementary Information:**

The online version contains supplementary material available at 10.1007/s12311-023-01526-4.

## Introduction

Stroke is a frequent neurological disorder and a major cause of disability worldwide [1]. Many stroke survivors experience problems with their hand motor function, which imposes a challenge for regaining a self-determined life [2]. An important and current rehabilitation strategy for alleviating constraints in hand motor function is task-specific training, i.e., the repeated, challenging practice of functional, goal-directed activities [3]. An important substrate of the gained behavioral improvement is training-induced plasticity within the underlying brain network [4, 5]. Core components of this network are the cortico-striatal and cortico-cerebellar systems and their dynamic interactions, which shape the evolution of novel or re-acquired motor memory traces [6–8].

One experimental approach that allows to further investigate and potentially support motor learning processes is the application of non-invasive brain stimulation (NIBS) in conjunction with behavioral training [9–14]. For example, it has been demonstrated that NIBS of the primary motor cortex (M1), which is applied during hand-based motor training, can improve the learning capacity [9, 10, 14]. The approach has gained broad interest in translational neuroscience research and has been investigated in several follow-up studies, for review see, e.g., Wessel et al. [15]. However, the strategy has so far yielded mixed results with variable response rates within and across studies, making it difficult to predict stimulation effects on the level of the individual patient [12, 16]. A convincing clinical translation of the conventional M1-based stimulation strategies has not been achieved yet [17]. A clear limitation of previous work is that, for the most part, the dynamic functional brain network-based architecture, necessary for mediating motor learning processes, has not been adequately considered. In other words, conventional strategies were largely based on a “one-node-only” stimulation approach.

To address this ongoing research challenge, we evaluated a sequential “dual-node” stimulation strategy focusing on the cortico-cerebellar system and characterized its impact on different subcomponents of learning. The model was chosen based on initial reports of successful modulation of motor learning processes through monofocal cerebellar (CB) NIBS in young healthy subjects [18, 19], the intactness of the target region in the majority of strokes cases (“non-lesioned entry to the system”) and the involvement of the cortico-cerebellar loop in stroke-related pathophysiological processes [20].

In the present study, we compared a sequential multifocal stimulation approach (stimulation sequence: M1-CB-M1-CB) to a monofocal control condition (stimulation sequence: M1-sham-M1-sham). The stimulation protocol was applied during a hand motor training and distributed over four training sessions on two consecutive days (D1S1-D1S2-D2S1-D2S2).

We hypothesized that the application of sequential multifocal facilitatory stimulation of the cortico-cerebellar loop would boost motor behavior and learning with respect to a monofocal control condition. Furthermore, multimodal data, including clinical and paired-pulse transcranial magnetic stimulation (ppTMS [21–24]), were acquired to search for features that allow to characterize stimulation response variability.

## Methods

### Participants

Twelve chronic stroke survivors were recruited for the study. One subject dropped out of the study after the screening session due to scheduling difficulties. The demographic characteristics of the remaining subjects, who participated in the study, are listed in Table 1.

**Table 1:** Patient characteristics. MCA (middle cerebral artery), FMA-UE (Fugl-Meyer Assessment Upper Extremity), NIHSS (National Institute of Health Stroke Scale), MMSE (Mini-Mental State Examination), SIS (Stroke Impact Scale), MEP (presence of stable upper limb motor evoked potentials recorded from the affected limb)

#	Lesion location	Time since stroke [months]	Gender	Age [years]	FMA-UE [max. 66]	NIHSS [max. 42]	MMSE [max. 30]	SIS [max. 100]	MEP
01	Paramedian pontine left	22	M	75	65	0	30	87	Yes
02	MCA deep branches right	37	F	74	59	1	28	61	No
03	MCA inferior division right	53	M	82	59	2	28	79	Yes
04	MCA frontal operculum, insula, temporo-parietal right	44	F	77	44	2	29	86	Yes
05	Paramedian pontine left	48	M	60	65	0	29	69	Yes
06	MCA frontal operculum right	97	F	72	61	0	29	83	Yes
07	MCA left	14	M	72	58	2	27	68	Yes
08	MCA deep branches left	66	M	61	40	1	28	67	No
09	MCA left	83	M	63	62	1	30	65	Yes
10	MCA right	126	M	60	61	1	30	88	Yes
11	Thalamus right	13	F	72	61	1	29	64	Yes
Mean	n/a	54.8	4/11 F	69.8	57.7	1.0	28.8	74.3	9/11 Yes
SD	n/a	35.7	n/a	7.6	8.1	0.8	1.0	10.4	n/a

The inclusion criteria were: ≥ 18 years of age, first ever stroke, ≥ 6 months post stroke, motor deficit, normal values of Mini-mental state examination (> 26/30), and absence of contraindication for NIBS. The exclusion criteria were: limited capacity to consent, multiple clinical apparent strokes, cerebellar stroke, concurrent neuropsychiatric diseases, history of seizures, intake of medication that potentially interacts with NIBS, high degree of spasticity (Ashworth > 2), musculoskeletal dysfunction that compromised finger movement, pregnancy, professional musicians or intense professional usage of a computer keyboard, intake of narcotic drugs, request of not being informed in case of incidental findings.

### Experimental Design

**Fig. 1:**
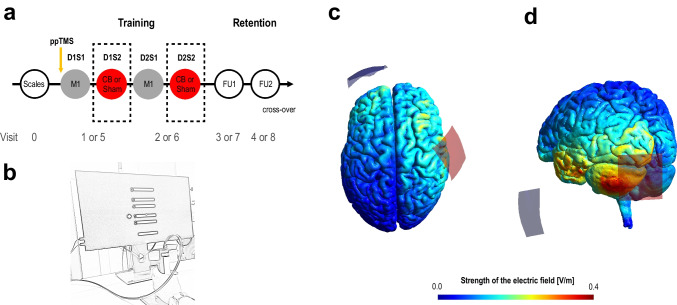
Experimental setup. **a** Timeline of the experiment. During visit 0 subjects’ baseline characteristics were assessed by applying a set of questionnaires and clinical scales (for details, see Table 1). In visits 1 and 2, respectively 5 and 6, the subjects conducted a motor training in two sessions per visit. Task retention was evaluated after 1 and about 10 days (visits 3 and 4, respectively 7 and 8 after the cross-over). **b** Illustration of the sequential grip force modulation task. The subjects had to navigate a cursor to target zones via the modulation of grip force using their affected hand. The order of targets followed a pre-defined sequence. **c**, **d** Depiction of the utilized tDCS montages, M1 stimulation (**c**), CB stimulation (**d**), and respective electric field simulations implemented in the SimNIBS platform [25]. The montage depicted illustrates the setup for a patient with a right hemispheric lesion

The study followed a randomized, double-blind, sham-controlled, cross-over design. The following two experimental conditions were tested and compared: (i) sequential multifocal stimulation following the stimulation sequence M1-CB-M1-CB (*in the following specified as MF-stimulation*) and (ii) a monofocal control condition respecting the sequence M1-sham-M1-sham (*in the following specified as Control*). In general, the subjects participated in 9 visits. For an illustration of the timeline, please see Fig. 1a. During visit 0, after obtaining informed consent, the stroke survivors were characterized by conducting a set of scores and scales, for details see Table 1. At the beginning of visit 1 an electrophysiological baseline assessment was conducted utilizing ppTMS techniques (see below). Afterwards, during visits 1 and 2, the stroke survivors conducted a motor training of two sessions on each of two consecutive days (D1S1, D1S2, D2S1, D2S2). There was a break of about 90 min between the training sessions within a day. Motor task retention was assessed at the follow-up visits (FU1, FU2) 1 day and about 10 days after the training phase. This was followed by a washout phase of an average of 35 (range 13-51) days and a cross-over to the other experimental condition. After the cross-over, visits 1 to 4, were repeated and labeled as sessions 5 to 8.

### Motor Learning Task

A computerized sequential grip force modulation task (SGFMT) served as the motor learning task, for further details please see Wessel and Draaisma et al. [26] and Fig. 1b. Prior to the baseline assessments and training, the subjects were briefed on the task procedures both verbally and in writing and conducted a simplified familiarization version of the task. The subjects were instructed to conduct the task as quickly and as accurately as possible. During the task, the participants had to control a grip-force sensor (*Current designs, Inc., Philadelphia, PA, USA*) with their paretic hand with the aim to navigate an on-screen cursor between a home zone and five target zones via modulation of their grip force. The force range was scaled to the individual maximum grip force. The order of the targets followed two pseudorandom, complexity-matched sequences A and B, which were randomized across subjects and stimulation conditions. The task difficulty was adjusted to the subject’s pre-baseline performance level. The subjects were allocated to the respective difficulty level based on the number of correctly performed sequences in a pre-baseline session (< 1: easy, 1: moderate, > 1: difficult task version). The pre-baseline block was followed by a 90 s baseline block at the allocated difficulty level to which all the subsequent motor learning data were corrected (see data processing below). The actual training consisted of 9 blocks of 90 s each separated by a 45 s break. In block 5 an additional pseudorandom, complex-matched target sequence was tested to assess for potential effects on sequence-independent motor performance. During the behavioral follow-up sessions, the subjects performed the motor task for 3 blocks, which were also separated by 45 s breaks.

### tDCS Protocol

NIBS was applied employing the transcranial direct current stimulation (tDCS) technique. The currents for tDCS were generated via a DC-stimulator plus (*neuroConn GmbH, Ilmenau, Germany*) and applied transcranial via rectangular (5 × 5 cm) sponge-like electrodes soaked in a saline solution that contained an electrode pad made of conductive rubber. The stimulation protocols were designed to modulate neuronal activity in the cortico-cerebellar system and were adopted from our prior work [13]. The active M1 stimulation protocol was defined by the following parameters: polarity, anodal stimulation; intensity, 1 mA; duration, 20 min; fade-in/out interval, 8 s; target electrode, TMS M1 hotspot contralateral to the affected hand, frontal edge oriented 45° to the midsagittal line; return electrode, supraorbital region ipsilateral to affected hand [27], for a depiction of the montage see Fig. 1c. The active CB stimulation protocol was defined by the following parameters: polarity, anodal stimulation; intensity, 2 mA; duration, 20 min; fade-in/out interval, 8 s; target electrode, 3 cm lateral of the inion over the CB ipsilateral to the affected hand; return electrode, over ipsilateral buccinator muscle [19, 28], for a depiction of the montage see Fig. 1d. The sham stimulation was applied in the cerebellar stimulation configuration using the same stimulation parameters as indicated in the active CB stimulation protocol, except that the current was already ramped down after 30 s of stimulation [19, 28].

### ppTMS

Paired-pulse transcranial magnetic stimulation (ppTMS) was utilized to assess GABAergic and glutamatergic neurotransmission linked to M1 at baseline [22]. The procedures are described in detail in our prior work [26, 29]. In brief, a MagPro X100 stimulator connected to an MC-B70 coil (*MagVenture, Farum, Denmark*) was used to deliver monophasic TMS pulses with posterior to anterior current direction in the underlying brain tissue. The coil was placed on the motor hot spot contralateral to the affected hand and oriented so that the handle pointed backward with an approximate angle of 45 degrees to the midsagittal line. The coil positioning was guided using a neuronavigation system (*Localite, Bonn, Germany*). Specifically, we assessed short intracortical inhibition at rest (SICI) and intracortical facilitation at rest (ICF) [22]. The motor-evoked potential (MEP) data were sampled from the first dorsal interosseous muscle (FDI) using a belly-tendon montage. The test pulse (TP) was adjusted to elicit a MEP of approximately 1 mV in the relaxed FDI. The conditioning pulses (CP) were adjusted to 80% of the resting-motor threshold (RMT), defined as the lowest stimulus intensity which produced a MEP with a peak-to-peak amplitude ≥ 50 μV in 5 out of 10 consecutive trials. SICI was evaluated at an inter-stimulus interval of 3 ms and ICF of 10 ms. For SICI and ICF procedures, please see prior work [26, 29].

### Data Processing

**Fig. 2:**
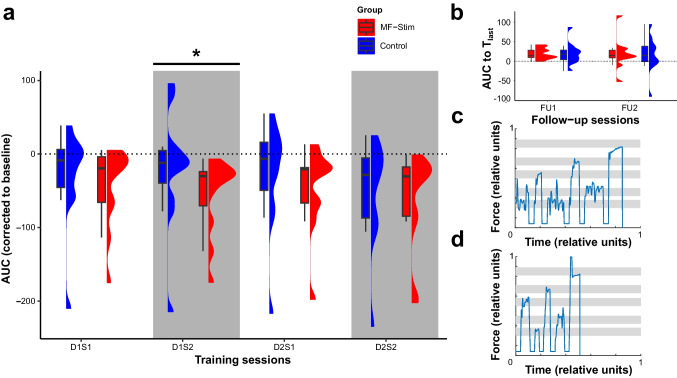
Results of behavioral training. **a** Training sessions separated by stimulation group. In the MF-stimulation (“MF-Stim”) condition, the stimulation sequence followed the order of active-M1, active-CB, active-M1, active-CB and was applied during the four consecutive training sessions (D1S1, D1S2, D2S1, D2S2). During the control condition (“Control”) the stimulation sequence was active-M1, sham-CB, active-M1, sham-CB. The grey background delineates the CB-stimulation sessions. More negative values indicate better performance. *A significant difference between the stimulation groups (*p* = .002). **b** Results of the follow-up sessions after 1 and about 10 days after the last training session. **c**, **d** Individual movement trajectory of one patient, who completed one sequence during the early stage (**c**) or during a later stage (**d**) of the training phase. For an additional figure depicting the individual data points per subject, please see the supplementary material Fig. S1

The behavioral motor learning data were sampled and pre-processed via custom written MATLAB scripts (*The MathWorks, Inc., Natick, MA, USA*). The a priori defined motor performance metric was the area under the curve (AUC) of the movement trajectory of correctly performed trials [26], for an illustration of an exemplary trajectory see Fig. 2c, d. For further analysis, the motor learning data were averaged per block. The motor learning score per block was corrected by subtraction to baseline.

The MEP data inspection and processing were performed offline using a custom MATLAB-based graphical user interface, which automatically checked for rejection criteria. The final decision for rejected trials had to be manually confirmed by the investigator. All trials were visually inspected. Trials were excluded when (1) the root mean square (RMS) value of the baseline EMG activity (100–0 ms before TMS pulse) was outside the mean ± 2 SD of all stimuli [30]; (2) MEP amplitude smaller than 50 μV, except for the SICI trials. The MEP amplitude was quantified by its peak-to-peak value measured in the time window of 20–50 ms after stimulation. The modulation of the ppTMS conditions was calculated as a percentage of the TP only condition and defined as: *mean (conditioned MEPs) / mean (unconditioned MEPs) × 100*.

### Statistical Analysis

The statistical analyses were conducted using RStudio (*version 1.4.1717*) and JASP (*version 0.16.4*). The normality of data was visually checked with Q-Q plots and histograms of residual values and further verified by the assessment of their skewness ranging between − 1 and 1 [31]. Statistical significance was assumed at p-values < .05. The data were analyzed using linear mixed-effects models included in the “lmerTest” package in RStudio [32]. Omnibus tests were performed with type II ANOVA of the model. Effect sizes were calculated as partial eta squared employing the “effectsize” package [33]. Post-hoc analyses were done by pairwise comparisons using the “emmeans” package (https://github.com/rvlenth/emmeans). Simple two-group comparisons have been analyzed using a paired samples t-test, Bonferroni corrected. The motor learning data were analyzed using the mean AUC output as the dependent variable and the training sessions (D1S1, D1S2, D2S1, and D2S2) and the stimulation conditions (MF-stimulation vs. Control) as the independent variable. To conduct a responder analysis, subgroups of the preselected metrics (baseline task performance (motor ability), SICI, ICF), were derived by a median split procedure. To evaluate for potential carry-over effects, the baseline evaluations and the slope of linear regression lines fitted through the training data were compared contrasting the before to the after cross-over phase separated per stimulation condition using a non-parametric (data not normally distributed) frequentist and Bayesian independent Mann-Whitney *U* test.

The data were visualized using raincloud plots, which were implemented employing the “ggplot2” package (https://github.com/tidyverse/ggplot2). In addition to a boxplot, the half-density distributions of the data are shown. For boxplots, the horizontal line corresponds to the median, the box limits to the 25th and 75th percentile of the interquartile range and the whiskers to the smallest/largest value within 1.5 times interquartile range below/above the box limits. For the figures, block data were averaged per session.

## Results

### Effect of CB-tDCS on Motor Behavior During the Training Phase

The analysis of the training sessions (D1S1, D1S2, D2S1, D2S2) indicated a significant main effect of STIMULATION F(1, 616.1) = 15.53, *p* < .001, η_p_^2^ = .02 and of SESSION F(3, 616.2) = 3.83, *p* = .01, η_p_^2^ = .02, but no STIMULATION × SESSION interaction *F*(3, 616.1) = 1.82, *p* = .142, η_p_^2^ = .01. Specifically, during MF-stimulation, the subjects demonstrated a globally better motor performance (smaller AUC of the movement trajectory for correct trials) in comparison to control (Fig. 2a). To further quantify the effects of the study intervention, in the next step, we analyzed the sessions with cerebellar stimulation (active vs. sham CB-tDCS) separately, namely D1S2 and D2S2. The results indicated a significant effect of STIMULATION F(1, 300.2) = 8.22, *p* = .004, η_p_^2^ =.03, of SESSION F(1, 300.3) = 7.79, *p* = .006, η_p_^2^ = .03 and a significant SESSION × STIMULATION interaction *F*(1, 300.3) = 5.27, *p* = .022, η_p_^2^ = .02. Post hoc pairwise comparisons indicated that the effect of STIMULATION was significant for SESSION D1S2 *t*(287.9) = 3.66, *p* = .002, but not for D2S2 *t*(286.7) = 0.4, *p* = .979, which suggests a learning-phase specific effect of CB-tDCS. Furthermore, the effect of SESSION was significant for the sham stimulation group *t*(49.2) = 3.69, *p* = .003, but not for the real stimulation group *t*(58.8) = 0.34, *p* = .986, indicating a CB stimulation effect during the early stages of motor learning that remains stable over time and an eventual “catching-up” in performance in the control group.

Retention was measured during two follow-up visits conducted 1 day and about 10 days after the training phase. The data were corrected by subtraction of the last block of the last training session to ensure a comparison of actual retention of the learned sequence with respect to the end of the training phase. Results showed no significant effect of STIMULATION F(1, 90.7) = 1.15, *p* = .286, η_p_^2^ = .04, or of FU F(1, 19.5) = 0.37, *p* = .55, η_p_^2^ = .002, or a STIMULATION × FU interaction F(1, 90.7) = 0.05, *p* = .827, η_p_^2^ = .001, see Fig. 2b.

To mitigate carry-over effects, after crossing over to the remaining stimulation condition, a wash-out phase was respected (mean: 35 days, range: 13 to 51 days). We were not able to detect differences for the baseline evaluation (MF-stimulation: *U* = 22.00, *p* = 0.25; Control: *U* = 18.00, *p* = 0.66) and the linear slopes (*U* = 11.00, *p* = 0.54 for both groups) fitted through the training data contrasting the before to the after cross-over phase. The complementary Bayesian analysis indicated that it was more likely that the baselines (MF-stimulation: BF01 = 1.44; Control: BF01 = 1.91) and slopes (MF-stimulation: BF01 = 1.70; Control: BF01 = 1.64) are equal than different. This makes a considerable carry-over effect unlikely. In addition to aggregated whole-group data, exemplary movement trajectories of a single participant sampled in the early and late training phase are depicted in Fig. 2c, d.

### Analysis of Temporal Subcomponents of Learning

To analyze offline learning, the within-day offline analysis was separated from the overnight offline analysis. This was done because of different stimulation paradigms (M1 vs. CB) and the additional factor of sleep during the overnight offline learning [34]. The analysis of the within-day offline learning between D1S1 and D1S2, and between D2S1 and D2S2 showed no significant effect of STIMULATION *F*(1, 33) = 0.002, *p* = .962, η_p_^2^ = .003, or of TIMING *F*(1, 33) = 0.095, *p* = .760, η_p_^2^ < .001, nor an interaction between STIMULATION × TIMING F(1, 29) = 0.27, *p* = .607, η_p_^2^ = .01. The overnight offline learning between session D1S2 and D2S1 showed no effect for STIMULATION *F*(1, 8.5) = 2.01, *p* = .192, η_p_^2^ = .19.

### Impact of Baseline Motor Ability on Stimulation Response

**Fig. 3:**
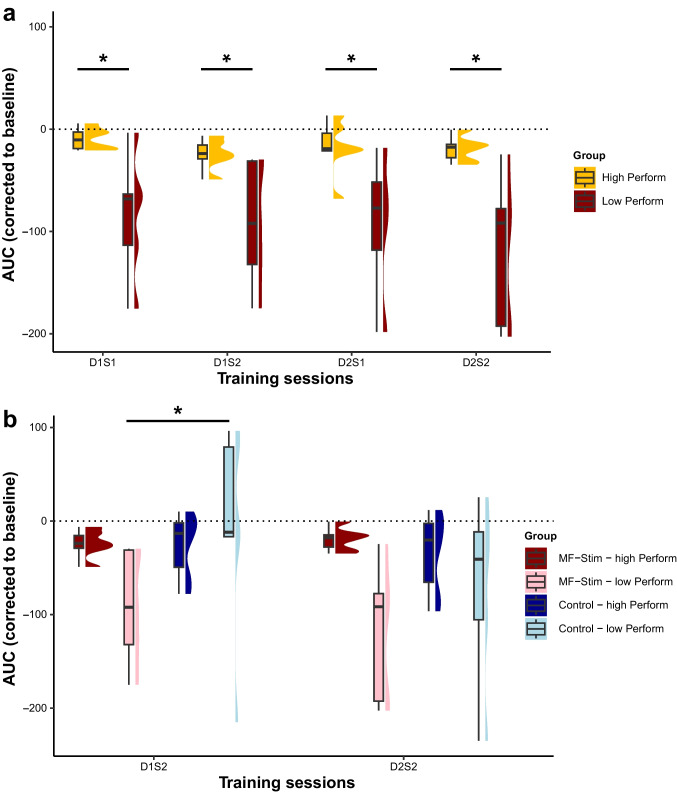
Motor ability-dependent effects of CB-stimulation. **a** The performance in the behavioral task during the active MF stimulation sessions only. The groups have been separated into high vs. low performer (“Perform”) groups based on the baseline performance. **b** The performance during the CB-stimulation sessions only. Groups are divided into MF-stimulation (“MF-Stim”) vs. control and high vs. low performance (“Perform”) during the preceding baseline session. *Significant difference between the respective contrast (*p* < .05). For an additional figure depicting the individual data points per subject, please see the supplementary material Fig. S2

The behavioral data of the training phase were separated by a median split into a low and high-performer group based on their baseline performance to investigate if motor ability at baseline impacts subjects’ response to MF-stimulation. The linear mixed-effects model included behavior (AUC) as the dependent variable. The independent variables were TIMING (D1S1, D1S2, D2S1, D2S2) and PERFORMANCE (high vs. low). The results showed a significant main effect for TIMING *F*(3,32.7) = 4.18, *p* = .013, η_p_^2^ = .28, for PERFORMANCE *F*(1, 10.9) = 10.14, *p* = .009, η_p_^2^ = .48 and a trend for an interaction between TIMING × PERFORMANCE *F*(3, 270.7) = 2.52, *p* = .059, η_p_^2^ = .03. Post hoc pairwise comparisons showed that MF-stimulation resulted in a stronger enhancement of motor behavior in the low performer group compared with the high performers in training session D1S1 *t*(15.4) = 2.59, *p* = .02, in D1S2 *t*(15.7) = 2.26, *p* = .038, in D2S1 *t*(15.9) = 3.13, *p* = .006 and D2S2 *t*(15.8) = 3.2, *p* = .006. This points towards an ability dependence of the induced stimulation effect, please see Fig. 3a.

To further explore the stimulation sensitivity, the active vs. sham stimulation conditions during the CB-stimulation sessions (D1S2, D2S2) were compared. The high vs. low performers were related to their respective stimulation conditions, creating four separate groups for comparison: “MF-Stim–high Perform,” “MF-Stim–low Perform,” “Control–high Perform,” and “Control–low Perform”. The results showed a significant main effect for TIMING *F*(1, 303.3) = 9.64, *p* = .002, η_p_^2^ = .03 and for GROUPS *F*(3, 27.6) = 14.17, *p* < .001, η_p_^2^ = .61, and an interaction effect for TIMING × GROUPS *F*(3, 393.4) = 5.49, *p* = .001, η_p_^2^ = .05. There was a significant difference in behavioral performance comparing MF-stimulation to control stimulation in the low performer group during D1S2 *t*(297.9) = − 6.25, *p* < .001; this effect did not remain in D2S2 *t*(297.4) = − 2.37, *p* = .085. However, there was no effect of MF-stimulation vs. control stimulation in the high performers during D1S2 *t*(295) = 0.45, *p* = .970 and during D2S2 *t*(295.9) = 1.48, *p* = .452. This indicates that the observed CB-tDCS effect during the early training phase on the group level was driven by a high stimulation protocol susceptibility for subjects with a lower baseline motor ability, please see Fig. 3b.

### Impact of Intracortical Inhibition and Facilitation of the Motor Cortex and Stimulation Response

The TMS-based metrics measured at the beginning of D1S1 showed no significant differences for the TP peak-to-peak amplitudes, the percentage of maximal stimulator output required to obtain adjusted TPs, SICI, or ICF between the before and after cross-over visits; for details, please see Table 2. This points towards a reliable adjustment of the TMS parameters, which assured that the metrics were obtained at a comparable range of the respective recruitment curves.

**Table 2:** Overview of the achieved adjustment for the TMS parameters. Table shows the mean and standard error of mean (SEM) in brackets of the different TMS parameters before and after cross-over. Paired samples *t*-test comparisons between before and after cross-over sessions are shown in the statistics column

Parameter	Before cross-over	After cross-over	Statistics
TP_only_ MEP Peak-to-peak amplitude (mV)	0.59 (0.08)	0.50 (0.09)	*t*(7) = − 1.80, *p* = .116
TP_only_ MEP Maximal stimulator output (%)	71.25 (5.66)	72.88 (5.30)	*t*(7) = 1.24, *p* = .256
SICI	79.65 (20.11)	81.52 (19.69)	*t*(7) = − 0.24, *p* = .815
ICF	142.32 (24.91)	139.04 (20.90)	*t*(7) = 0.30, *p* = .771

To evaluate if the assessed metrics contain information that determines the subsequent response to stimulation, the data were divided into two groups based on a median split. Only the CB-stimulation sessions (D1S2, D2S2) were considered for the analysis. Following this procedure, we obtained two subgroups per assessed TMS metric, weak vs. strong inhibition for SICI and weak vs. strong facilitation for ICF. These factors were grouped based on the stimulation condition, resulting in four groups: “MF-Stim–strong SICI or ICF”; “MF-Stim–weak SICI or ICF”; “Control–strong SICI or ICF”; “Control–weak SICI or ICF”.

**Fig. 4:**
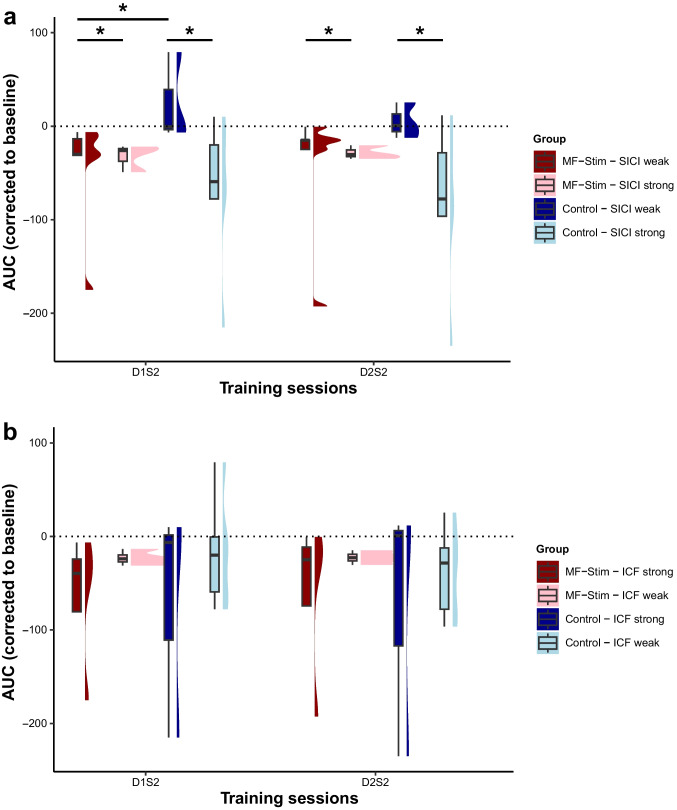
Relationship of ppTMS-derived metrics and stimulation response. Groups were separated based on the level of inhibition for SICI, respectively facilitation for ICF and applied stimulation condition: MF-Stimulation (“MF-Stim”) vs. control stimulation (“Control”). Only the sessions, in which active CB-stimulation or sham was applied (D1S2 or D2S2), were considered. **a** Baseline SICI strong vs. weak inhibition in relation to task performance. **b** Baseline ICF strong vs. weak facilitation in relation to task performance. *Significant difference between the respective contrast (*p* < .05). For an additional figure depicting the individual data points per subject, please see the supplementary material Fig. S3

For SICI the results indicated a significant main effect for TIMING F(1, 222) = 5.35, *p* = .022, η_p_^2^ = .02, and for GROUPS F(3, 224.9) = 25.29, *p* < .001, η_p_^2^ = .25. There was no interaction between TIMING × GROUPS *F*(3, 222) = 1.19, *p* = .314, η_p_^2^ = .02. Post hoc pairwise comparisons showed a better performance for strong vs. weak inhibition with MF-stimulation during session D1S2 *t*(219.8) = 5.69, *p* < .001 and D2S2 *t*(220.4) = 4.63, *p* < .001. There was better performance for strong vs. weak inhibition with control stimulation during D1S2 *t*(222.9) = 7.4, *p* < .001, and D2S2 *t*(222.6) = 6.84, *p* < .001. The participants with weak inhibition performed significantly better with MF-stimulation vs. control stimulation during D1S2 *t*(220.3) = − 3.56, *p* = .011, but not during D2S2 *t*(218.4) = − 2.61, *p* = .159. There was no difference between MF-stimulation vs. control stimulation in the strong inhibition group during D1S2 *t*(215.2) = − 1.29, *p* = .903 or during D2S2 *t*(215.2) = 0.7, *p* = .997, please see Fig. 4a.

The ICF results showed a significant main effect for TIMING *F*(1, 221.9) = 5.55, *p* = .019, η_p_^2^ = .02 and for GROUPS *F*(3, 208.6) = 3.23, *p* = .024, η_p_^2^ = .04, but not an interaction between TIMING × GROUPS *F*(3, 221.9) = 1.37, *p* = .252, η_p_^2^ = .02. On visual inspection, there seemed to be an indication that the participants with strong facilitation perform better than the participants with weak facilitation in the MF-stimulation but not the control condition. However, post hoc comparisons demonstrated no significant differences between any of the groups or for any of the timings, please see Fig. 4b.

For an exploratory sub-analysis contrasting stimulation response based on the presence versus absence of stable upper limb motor evoked potentials (MEPs) of the affected limb, please see the supplementary material section “impact of corticospinal tract integrity on stimulation response” and Fig. S4. In brief, this exploratory analysis may indicate a higher stimulation response in stroke survivors with a no-MEP status.

## Discussion

The present study suggests that sequential multifocal tDCS of M1 and CB improved motor performance in a hand-based, sequential motor task in chronic stroke survivors. The effect was driven by active CB-stimulation during the first training day (D1S2), indicating stimulation efficacy during the early phase of learning. Furthermore, several features that were associated with the subjects’ stimulation response, such as baseline motor performance (motor ability), and level of SICI or CST integrity, were detected.

### CB-tDCS Boosts Motor Behavior in the Early Training Phase

Several neurobiological models have been developed in systems neuroscience to describe the involvement of distinct neuronal structures underlying the process of motor skill learning [6–8, 35]. The core assumptions are that the cortico-striatal and the cortico-cerebellar system represent crucial neural substrates and that the engagement of the different subregions is learning phase-dependent. In addition, an intrinsic, phase-dependent shift of neural representations has been described for the targeted cerebellum. For instance, Doyon and colleagues were able to characterize the evolution of the activation pattern during a motor sequence learning task within the cerebellum by using functional magnetic resonance imaging [36]. The pattern is characterized by a pronounced activation of the cerebellar cortex during the early learning stage. As learning progressed, the level of cortical activation decreased, and activity at the dentate nucleus level increased. Moreover, the shift of engagement from the cortex to the level of the deep nuclei is supported by theoretical circuit-based models of cerebellum-mediated motor learning. For example, Mauk predicted a sequence of distributed plasticity across the cerebellar circuitry [37]. The model suggests that during the early learning phase plasticity mainly occurs at the parallel fiber-Purkinje cell synapses in cortex. The paired presentation of a respective sensory context (mossy fibers) and error signals (climbing fibers) during the learning process induces postsynaptic long-term depression (LTD) and subsequently a disinhibition of Purkinje cells’ output. This in turn results in long-term potentiation (LTP) at mossy fiber-nucleus cell synapses at the later stage.

We speculate that anodal CB-tDCS application may have supported these inherent processes through the promotion of LTD-like plasticity at the early learning state, namely the D1S2 session [38–40] (see also Fig. 2a). Conversely at a later stage of learning (D2S2), a major part of the underlying plasticity was already transferred to deeper cerebellar structures and other systems, which were not directly targeted, thus most likely not sufficiently modulated by the CB-tDCS protocol. A complementary explanation for the phase-specific CB-tDCS effect could be that learning in the early training phase largely relied on an error-based mechanism, which is driven by the mismatch of intended and perceived motor outcome (sensory-prediction error) and which strongly involves the cerebellum [41]. It is possible that a stronger weight was set on other learning mechanisms at the later learning stage, which mainly recruited neuronal processing in other brain areas. For example, that rather reinforcement-based (basal ganglia), use-dependent (M1), or strategy-based learning (prefrontal cortex) processes were recruited. Thus, it can be speculated that these alternative learning mechanisms, which have a different brain topographical profile, were not responsive to CB-tDCS to a similar degree.

### No Effect of Multifocal M1-CB Stimulation on the Overall Training Success and Skill Retention

Current evidence suggests that different tDCS protocols exert their effects via the modulation of distinct temporal components of motor learning [12, 14]. For example, in their seminal work, Reis and colleagues could show that anodal tDCS applied to M1 during the acquisition of a task that required young healthy subjects to execute and learn a sequence of pinch forces was able to enhance the total learning with respect to a sham control and that this effect was driven by an enhancement of offline effects [9]. However, other studies emphasized online effects of anodal M1 tDCS protocols, when employing different motor learning paradigms [11, 42, 43]. Likewise, the most susceptible temporal components to CB-tDCS appear to be task-specific [18, 19]. Despite this apparent dependence of the most susceptible components on the applied task, the site of stimulation, and the cohort studied, an objective of the present study was to investigate whether the sequential reinforcement of different learning components through stimulation of different targets could increase the overall effect size. The present study was designed in a sequential fashion in the order M1- followed by CB-stimulation based on previous in-house data, which indicated that anodal M1-tDCS mainly exerted its effects via modulation of online and CB-tDCS mainly via the modulation of offline effects [11, 19]. We hypothesized that this sequential engagement of different mechanisms underlying motor skill learning boosts the overall training success by addressing a different aspect of the motor learning process. The acquired data did not validate this hypothesis. At the end of the training (D2S2 session), we were not able to detect differences across stimulation groups (see Fig. 2a). As discussed above the CB-tDCS effect was phase-specific and boosted primarily the performance in the early training phase (D1S2). This could be potentially explained by ceiling effects of the task and a consecutive catch-up of the performance of the control group. However, the participants of the active stimulation group were still able to improve on the task after D1S2 (mean ± SEM: − 55.18 ± 16.41) when tested at session D2S2 (mean ± SEM: − 64.11 ± 21.52), which argues against a pure ceiling effect. Finally, we did not detect stimulation-associated effects on task retention, which further strengthens the notion that the applied multifocal stimulation protocol was able to modulate single individual components of learning in the early training phase (enhanced motor performance during D1S2), but the “boosting” effects were not retained at later evaluations.

### Responder/Non-responder Analyses

Retrospective analyses revealed a high degree of response variability towards tDCS-aided motor learning-based interventions within and across studies in stroke survivors [44]. Several factors that have been associated with stimulation response, such as lesion location, time since stroke, or level of impairment have been identified; for further reading, see, e.g., Wessel et al. [20] and Wessel and Egger et al. [16]. Based on this emerging responder/non-responder pattern for tDCS protocols in general, we investigated the effects of a priori determined possible influencing factors of stimulation response.

In a first step, we investigated the effect of baseline motor performance (motor ability). This we approached by dividing the participants into a weak and a high performer group based on their baseline performance in the task. The analysis indicated that the CB-tDCS-mediated effects on the early learning phase were driven by a high susceptibility of participants with low baseline motor ability. A possible mechanism underlying this finding is that participants in the low-performer group, who performed less accurate movement trajectories, received a stronger error signal while conducting the task at the early learning stage. It is possible that this increased error signal, which was mediated by climbing fiber input and which was further processed at the level of the cerebellar cortex [45], may have provided a crucial point of action for CB-tDCS.

In a second step, we evaluated possible relationships of the tested ppTMS metrics, SICI and ICF at baseline, and stimulation response. We have looked exclusively at the CB-stimulation sessions to be able to compare active vs. sham stimulation conditions. Our findings indicated that strong SICI (inhibited state) was related to better performance, both during sham and active stimulation. Furthermore, our results indicated that active CB-stimulation significantly improved performance in participants with weak initial SICI levels (disinhibited state) compared to sham stimulation (see Fig. 4a). At first sight, this seems to be in contrast to a previous study showing a relationship between higher GABA levels (more inhibited state) and better performance in response to anodal tDCS of M1 [46]. Furthermore, the result challenges available electrophysiological models linking the cerebellar output tone (quantified via the TMS-based assessment of cerebellum brain inhibition (CBI), for details see [47]) with the state of SICI in the motor cortex [48]. This model suggests that a stronger CBI is related to weaker SICI values (disinhibition). Strengthening of CBI, which would be the most likely consequence of anodal CB-tDCS, would not impact on a system, which does not have sufficient room for further disinhibition. These considerations render it unlikely that the CB-tDCS induced effect was to a significant extent mediated via modulation of SICI in the motor cortex. The ICF measurements at rest showed more variable results, which could not be distinguishably related to motor performance or stimulation effects. This is comparable to previous findings for ICF during a similar task in healthy young adults [26].

### Limitations and Future Perspective

The present study has some limitations. Firstly, the sample size is rather small for statistical comparisons. Yet, the sample size is within the range of other proof-of-principle NIBS studies investigating effects on motor function and related mechanisms in stroke survivors [15, 49–51]. Moreover, a cross-over design was used to increase statistical power. Secondly, one of the main limitations of conventional tDCS is the lack of focality of stimulation. Due to the relatively large electrode sizes, the electric field is more dispersed [52] (see also Fig. 1c, d). It might well be that adjacent brain areas have been simultaneously stimulated. Different shapes of electrodes such as concentric electrodes, which have been shown to increase focality, could be used in future studies [52]. Thirdly, a further limitation of the study is that we did not include a control condition with active stimulation of a functionally not relevant brain site in addition to the employed well-established sham control. The global amount of electrical charge passed to the brain was different across experimental conditions. However, based on literature testing active stimulation of control sites, e.g., [53, 54], it is highly unlikely that a global unspecific stimulation will be the driver of the observed learning-phase specific neuromodulation effect. Fourthly, it could be that the difficulty level of the task has an influence on the individual outcome. Although the task was adjusted to the baseline performance level of each participant, there is a considerable difference in task performance between low and high performers measured at baseline. This could point towards a ceiling effect of high-performing participants. Moreover, the task has more temporal restrictions than other motor tasks such as the sequential finger tapping task. The grip force modulation task required the participants to hold force and remain in the target for a specific amount of time before returning to the home zone. This results in a limited possibility to increase speed during the task, which may have reduced the possibility to improve. However, using tests like the sequential finger tapping tests might result in a skewed image of motor learning in stroke survivors as only very mildly impaired patients would be able to perform the task. Therefore, using grip force modulation, which comprises more gross movements, allows testing sequence learning in a larger variety of stroke patients. As discussed above, there was a considerable variability in baseline task performance (motor ability) across patients. This might impact on the evolution of the subsequent learning trajectories. To mitigate this bias, we employed a baseline correction and employed a cross-over design that patients could “serve as their own controls.” Lastly, another limitation lies within the current study design. To reduce the amount of time to acquire the data and to not make the experiment too lengthy and straining for the patients, we decided not to measure SICI and ICF values post-training. However, in light of the current results, we can argue that the additional TMS measures would have been informative. Future studies should consider including post-training TMS measurements while considering the time and comfort of the patients.

## Conclusion

In summary, the present results indicate that it is possible to modulate hand motor performance of chronic stroke survivors through CB-tDCS application. The effect was driven by a selective enhancement of task performance in the early training phase. The subsequently conducted responder analyses indicated that stroke survivors with low baseline motor ability and a maintained state of motor cortical disinhibition in the chronic phase benefited the most from the intervention. Especially, these patients relied on error-based learning mechanisms, which have been linked to neuronal processing at the cerebellar cortex. It is of note that the CB-tDCS associated facilitation of behavior at the early training phase, did not translate into enhanced overall training success or skill retention, which could be related to ceiling effects of the applied motor learning task.

### Supplementary Information

Supplementary materials 1: Fig. S1 Results of behavioral training showing data points for individual subjects #01 to #11. • #01, • #02, • #03, • #04, • #05, • #06, • #07, • #08, • #09, • #10, • #11. (a) Training sessions separated by stimulation group. In the MF-stimulation (“MF-Stim”) condition, the stimulation sequence followed the order of active-M1, active-CB, active-M1, active-CB and was applied during the four consecutive training sessions (D1S1, D1S2, D2S1, D2S2). During the control condition (“Control”) the stimulation sequence was active-M1, sham-CB, active-M1, sham-CB. The grey background delineates the CB-stimulation sessions. More negative values indicate better performance. *: indicates a significant difference between the stimulation groups (p = .002). (b) Results of the follow-up sessions after 1 and about 10 days after the last training session. Individual movement trajectory of one patient, who completed one sequence during the early stage (c) or during a later stage (d) of the training phase. Fig. S2 Motor ability-dependent effects of CB-stimulation showing data points for individual subjects #01 to #11. • #01, • #02, • #03, • #04, • #05, • #06, • #07, • #08, • #09, • #10, • #11. (a) The performance in the behavioral task during the active MF-stimulation sessions only. The groups have been separated into high vs. low performer (“Perform”) groups based on the baseline performance. (b) The performance during the CB-stimulation sessions only. Groups are divided into MF-stimulation (“MF-Stim”) vs. control and high vs. low performance (“Perform”) during the preceding baseline session. *: indicates significant difference between the respective contrast (p < .05). Fig. S3 Relationship of ppTMS-derived metrics and stimulation response for individual subjects. • #01, • #03, • #04, • #05, • #06, • #07, • #09, • #10. Please note for subjects #02 and #08, we were not able to record motor evoked potentials (MEPs) from the affected limb of sufficient size. Subject #11 has MEPs of sufficient size. However, to adjust the protocol to the subject’s requests no SICI and ICF measurements were conducted. Groups were separated based on the level of inhibition for SICI, respectively facilitation for ICF and applied stimulation condition: MF-Stimulation (“MF-Stim”) vs. control stimulation (“Control”). Only the sessions, in which active CB-stimulation or sham was applied (D1S2 or D2S2), were considered. (a) Baseline SICI strong vs. weak inhibition in relation to task performance. (b) Baseline ICF strong vs. weak facilitation in relation to task performance. *: indicates significant difference between the respective contrast (p < .05). Fig. S4 Exploratory responder analysis based on the presence of MEPs (no vs. yes). Subjects #02 and #08 had a no-MEP status. Training performance is shown as AUC corrected to baseline. More negative values indicate better performance. The tDCS effect seemed to be pronounced for stroke survivors with a no-MEP status. Error bars correspond to standard error of the mean (SEM). (DOCX 443 kb)

## References

[1] GBD 2016 Neurology Collaborators (2019). Global, regional, and national burden of neurological disorders, 1990-2016: a systematic analysis for the Global Burden of Disease Study 2016. Lancet Neurol.

[2] Micera S, Caleo M, Chisari C, Hummel FC, Pedrocchi A (2020). Advanced neurotechnologies for the restoration of motor function. Neuron..

[3] Winstein CJ, Stein J, Arena R, Bates B, Cherney LR, Cramer SC (2016). Guidelines for adult stroke rehabilitation and recovery: a guideline for healthcare professionals from the American Heart Association/American Stroke Association. Stroke..

[4] Krakauer JW (2006). Motor learning: its relevance to stroke recovery and neurorehabilitation. Curr Opin Neurol..

[5] Hardwick RM, Rottschy C, Miall RC, Eickhoff SB (2013). A quantitative meta-analysis and review of motor learning in the human brain. Neuroimage..

[6] Hikosaka O, Nakamura K, Sakai K, Nakahara H (2002). Central mechanisms of motor skill learning. Curr Opin Neurobiol..

[7] Doyon J, Benali H (2005). Reorganization and plasticity in the adult brain during learning of motor skills. Curr Opin Neurobiol..

[8] Dayan E, Cohen LG (2011). Neuroplasticity subserving motor skill learning. Neuron..

[9] Reis J, Schambra HM, Cohen LG, Buch ER, Fritsch B, Zarahn E (2009). Noninvasive cortical stimulation enhances motor skill acquisition over multiple days through an effect on consolidation. Proc Natl Acad Sci U S A..

[10] Zimerman M, Heise KF, Hoppe J, Cohen LG, Gerloff C, Hummel FC (2012). Modulation of training by single-session transcranial direct current stimulation to the intact motor cortex enhances motor skill acquisition of the paretic hand. Stroke..

[11] Zimerman M, Nitsch M, Giraux P, Gerloff C, Cohen LG, Hummel FC (2013). Neuroenhancement of the aging brain: restoring skill acquisition in old subjects. Annals of neurology..

[12] Buch ER, Santarnecchi E, Antal A, Born J, Celnik PA, Classen J (2017). Effects of tDCS on motor learning and memory formation: A consensus and critical position paper. Clin Neurophysiol..

[13] Wessel MJ, Park C-H, Beanato E, Cuttaz EA, Timmermann JE, Schulz R (2021). Multifocal stimulation of the cerebro-cerebellar loop during the acquisition of a novel motor skill. Sci Rep..

[14] Maceira-Elvira P, Timmermann JE, Popa T, Schmid A-C, Krakauer JW, Morishita T (2022). Dissecting motor skill acquisition: Spatial coordinates take precedence. Sci Adv..

[15] Wessel MJ, Zimerman M, Hummel FC (2015). Non-invasive brain stimulation: an interventional tool for enhancing behavioral training after stroke. Front Hum Neurosci..

[16] Wessel MJ, Egger P, Hummel FC (2021). Predictive models for response to non-invasive brain stimulation in stroke: A critical review of opportunities and pitfalls. Brain Stimul..

[17] Lefaucheur J-P, Antal A, Ayache SS, Benninger DH, Brunelin J, Cogiamanian F (2017). Evidence-based guidelines on the therapeutic use of transcranial direct current stimulation (tDCS). Clin Neurophysiol..

[18] Cantarero G, Spampinato D, Reis J, Ajagbe L, Thompson T, Kulkarni K (2015). Cerebellar direct current stimulation enhances on-line motor skill acquisition through an effect on accuracy. J Neurosci..

[19] Wessel MJ, Zimerman M, Timmermann JE, Heise KF, Gerloff C, Hummel FC (2016). Enhancing consolidation of a new temporal motor skill by cerebellar noninvasive stimulation. Cereb Cortex..

[20] Wessel MJ, Hummel FC (2018). Non-invasive cerebellar stimulation: a promising approach for stroke recovery?. Cerebellum..

[21] Kujirai T, Caramia MD, Rothwell JC, Day BL, Thompson PD, Ferbert A (1993). Corticocortical inhibition in human motor cortex. J Physiol..

[22] Chen R (2004). Interactions between inhibitory and excitatory circuits in the human motor cortex. Exp Brain Res..

[23] Hummel FC, Steven B, Hoppe J, Heise K, Thomalla G, Cohen LG (2009). Deficient intracortical inhibition (SICI) during movement preparation after chronic stroke. Neurology..

[24] Liuzzi G, Hörniß V, Lechner P, Hoppe J, Heise K, Zimerman M (2014). Development of movement-related intracortical inhibition in acute to chronic subcortical stroke. Neurology..

[25] Thielscher A, Antunes A, Saturnino GB. Field modeling for transcranial magnetic stimulation: a useful tool to understand the physiological effects of TMS? 2015 37th Annual International Conference of the IEEE Engineering in Medicine and Biology Society (EMBC) [Internet]. Milan: IEEE; 2015 [cited 2021 Nov 15]. p. 222–5. Available from: http://ieeexplore.ieee.org/document/7318340/10.1109/EMBC.2015.7318340 26736240

[26] Wessel MJ, Draaisma LR, de Boer AFW, Park C-H, Maceira-Elvira P, Durand-Ruel M (2020). Cerebellar transcranial alternating current stimulation in the gamma range applied during the acquisition of a novel motor skill. Sci Rep..

[27] Hummel F, Celnik P, Giraux P, Floel A, Wu W-H, Gerloff C (2005). Effects of non-invasive cortical stimulation on skilled motor function in chronic stroke. Brain..

[28] Galea JM, Jayaram G, Ajagbe L, Celnik P (2009). Modulation of cerebellar excitability by polarity-specific noninvasive direct current stimulation. J Neurosci..

[29] Wessel MJ, Draaisma LR, Morishita T, Hummel FC (2019). The Effects of stimulator, waveform, and current direction on intracortical inhibition and facilitation: a TMS comparison study. Front Neurosci..

[30] van de Ruit M, Grey MJ (2016). The TMS Map Scales with Increased Stimulation Intensity and Muscle Activation. Brain Topogr..

[31] Ryu E. Effects of skewness and kurtosis on normal-theory based maximum likelihood test statistic in multilevel structural equation modeling. Behav Res Methods. 2011;43:1066–74. 10.3758/s13428-011-0115-7 21671139

[32] Kuznetsova A, Brockhoff PB, Christensen RHB. lmerTest package: tests in linear mixed effects models. J Stat Softw. 2017;82:1–26.

[33] Ben-Shachar M, Lüdecke D, Makowski D. effectsize: estimation of effect size indices and standardized parameters. JOSS. 2020;5:2815.

[34] Walker MP, Brakefield T, Morgan A, Hobson JA, Stickgold R (2002). Practice with sleep makes perfect: sleep-dependent motor skill learning. Neuron..

[35] Doyon J, Gabitov E, Vahdat S, Lungu O, Boutin A (2018). Current issues related to motor sequence learning in humans. Current Opinion in Behavioral Sciences..

[36] Doyon J, Ungerleider LG. Functional anatomy of motor skill learning. Neuropsychology of memory, 3rd ed. New York, NY, US: The Guilford Press; 2002. p. 225–38.

[37] Mauk MD (1997). Roles of cerebellar cortex and nuclei in motor learning: contradictions or clues?. Neuron..

[38] Fritsch B, Reis J, Martinowich K, Schambra HM, Ji Y, Cohen LG (2010). Direct current stimulation promotes BDNF-dependent synaptic plasticity: potential implications for motor learning. Neuron..

[39] Ferrucci R, Priori A (2014). Transcranial cerebellar direct current stimulation (tcDCS): motor control, cognition, learning and emotions. NeuroImage..

[40] Rohan JG, Miklasevich MK, McInturf SM, Bechmann NA, Moore RJ, Hatcher-Solis C (2020). Polarity and subfield specific effects of transcranial direct current stimulation on hippocampal plasticity. Neurobiol Learn Mem..

[41] Spampinato D, Celnik P (2021). Multiple motor learning processes in humans: defining their neurophysiological bases. Neuroscientist..

[42] Nitsche MA, Schauenburg A, Lang N, Liebetanz D, Exner C, Paulus W (2003). Facilitation of implicit motor learning by weak transcranial direct current stimulation of the primary motor cortex in the human. Journal of cognitive neuroscience..

[43] Stagg CJ, Jayaram G, Pastor D, Kincses ZT, Matthews PM, Johansen-Berg H (2011). Polarity and timing-dependent effects of transcranial direct current stimulation in explicit motor learning. Neuropsychologia..

[44] Kang N, Summers JJ, Cauraugh JH (2016). Transcranial direct current stimulation facilitates motor learning post-stroke: a systematic review and meta-analysis. J Neurol Neurosurg Psychiatry..

[45] Ito M (2000). Mechanisms of motor learning in the cerebellum. Brain Res..

[46] O’Shea J, Boudrias M-H, Stagg CJ, Bachtiar V, Kischka U, Blicher JU (2014). Predicting behavioural response to TDCS in chronic motor stroke. NeuroImage..

[47] Ugawa Y, Uesaka Y, Terao Y, Hanajima R, Kanazawa I (1995). Magnetic stimulation over the cerebellum in humans. Annals of neurology..

[48] Daskalakis ZJ, Paradiso GO, Christensen BK, Fitzgerald PB, Gunraj C, Chen R (2004). Exploring the connectivity between the cerebellum and motor cortex in humans. J Physiol..

[49] Fregni F, Boggio PS, Mansur CG, Wagner T, Ferreira MJL, Lima MC (2005). Transcranial direct current stimulation of the unaffected hemisphere in stroke patients. Neuroreport..

[50] Madhavan S, Weber KA, Stinear JW (2011). Non-invasive brain stimulation enhances fine motor control of the hemiparetic ankle: implications for rehabilitation. Exp Brain Res..

[51] Stagg CJ, Bachtiar V, O’Shea J, Allman C, Bosnell RA, Kischka U (2012). Cortical activation changes underlying stimulation-induced behavioural gains in chronic stroke. Brain..

[52] Saturnino GB, Madsen KH, Siebner HR, Thielscher A (2017). How to target inter-regional phase synchronization with dual-site Transcranial Alternating Current Stimulation. NeuroImage..

[53] Nitsche MA, Paulus W (2000). Excitability changes induced in the human motor cortex by weak transcranial direct current stimulation. J Physiol..

[54] Galea JM, Vazquez A, Pasricha N, de Xivry J-JO, Celnik P (2011). Dissociating the roles of the cerebellum and motor cortex during adaptive learning: the motor cortex retains what the cerebellum learns. Cereb Cortex..

