# Supplementary material for: Multi-focal Stimulation of the Cortico-cerebellar Loop During the Acquisition of a Novel Hand Motor Skill in Chronic Stroke Survivors
Source: Cerebellum. 2023 Feb 18;23(2):341–54. doi: 10.1007/s12311-023-01526-4 (PMC10951005; doi:10.1007/s12311-023-01526-4)
Supplement: Supplementary file 1 — Fig. S1 Results of behavioral training showing data points for individual subjects #01 to #11. • #01, • #02, • #03, • #04, • #05, • #06, • #07, • #08, • #09, • #10, • #11. (a) Training sessions separated by stimulation group. In the MF-stimulation (“MF-Stim”) condition, the stimulation sequence followed the order of active-M1, active-CB, active-M1, active-CB and was applied during the four consecutive training sessions (D1S1, D1S2, D2S1, D2S2). During the control condition (“Control”) the stimulation sequence was active-M1, sham-CB, active-M1, sham-CB. The grey background delineates the CB-stimulation sessions. More negative values indicate better performance. *: indicates a significant difference between the stimulation groups (p = .002). (b) Results of the follow-up sessions after 1 and about 10 days after the last training session. Individual movement trajectory of one patient, who completed one sequence during the early stage (c) or during a later stage (d) of the training phase. Fig. S2 Motor ability-dependent effects of CB-stimulation showing data points for individual subjects #01 to #11. • #01, • #02, • #03, • #04, • #05, • #06, • #07, • #08, • #09, • #10, • #11. (a) The performance in the behavioral task during the active MF-stimulation sessions only. The groups have been separated into high vs. low performer (“Perform”) groups based on the baseline performance. (b) The performance during the CB-stimulation sessions only. Groups are divided into MF-stimulation (“MF-Stim”) vs. control and high vs. low performance (“Perform”) during the preceding baseline session. *: indicates significant difference between the respective contrast (p < .05). Fig. S3 Relationship of ppTMS-derived metrics and stimulation response for individual subjects. • #01, • #03, • #04, • #05, • #06, • #07, • #09, • #10. Please note for subjects #02 and #08, we were not able to record motor evoked potentials (MEPs) from the affected limb of sufficient size. Subject #11 has MEPs of sufficie [file 12311_2023_1526_MOESM1_ESM.docx]

**Supplementary material for**

**Multi-focal stimulation of the cortico-cerebellar loop during the acquisition of a novel hand motor skill in chronic stroke survivors**

Maximilian J. Wessel and Laurijn R. Draaisma *et al.*

Corresponding author: Friedhelm C. Hummel, friedhelm.hummel@epfl.ch

# 1. Behavioral training

## **Fig. S1 Results of behavioral training showing data points for individual subjects #01 to #11.** • #01, • #02, • #03, • #04, • #05, • #06, • #07, • #08, • #09, • #10, • #11. **(a)** Training sessions separated by stimulation group. In the MF-stimulation (“MF-Stim”) condition, the stimulation sequence followed the order of active-M1, active-CB, active-M1, active-CB and was applied during the four consecutive training sessions (D1S1, D1S2, D2S1, D2S2). During the control condition (“Control”) the stimulation sequence was active-M1, sham-CB, active-M1, sham-CB. The grey background delineates the CB-stimulation sessions. More negative values indicate better performance. *: indicates a significant difference between the stimulation groups (p = .002). **(b)** Results of the follow-up sessions after 1 and about 10 days after the last training session. Individual movement trajectory of one patient, who completed one sequence during the early stage **(c)** or during a later stage **(d)** of the training phase.

# 2. Motor ability-dependent effects of CB-stimulation

## **Fig. S2 Motor ability-dependent effects of CB-stimulation showing data points for individual subjects #01 to #11.** • #01, • #02, • #03, • #04, • #05, • #06, • #07, • #08, • #09, • #10, • #11. **(a)** The performance in the behavioral task during the active MF-stimulation sessions only. The groups have been separated into high vs. low performer (“Perform”) groups based on the baseline performance. **(b)** The performance during the CB-stimulation sessions only. Groups are divided into MF-stimulation (“MF-Stim”) vs. control and high vs. low performance (“Perform”) during the preceding baseline session. *: indicates significant difference between the respective contrast (p < .05).

# 3. Relationship of ppTMS-derived metrics and stimulation response.

## **Fig. S3 Relationship of ppTMS-derived metrics and stimulation response for individual subjects.** • #01, • #03, • #04, • #05, • #06, • #07, • #09, • #10. Please note for subjects #02 and #08, we were not able to record motor evoked potentials (MEPs) from the affected limb of sufficient size. Subject #11 has MEPs of sufficient size. However, to adjust the protocol to the subject’s requests no SICI and ICF measurements were conducted. Groups were separated based on the level of inhibition for SICI, respectively facilitation for ICF and applied stimulation condition: MF-Stimulation (“MF-Stim”) vs. control stimulation (“Control”). Only the sessions, in which active CB-stimulation or sham was applied (D1S2 or D2S2), were considered. **(a)** Baseline SICI strong vs. weak inhibition in relation to task performance. **(b)** Baseline ICF strong vs. weak facilitation in relation to task performance. *: indicates significant difference between the respective contrast (p < .05).

# 4. Impact of corticospinal tract integrity on stimulation response

As an exploratory sub-analysis, we separated the stroke survivors by their corticospinal tract (CST) integrity, quantified via the presence or non-presence of MEPs recorded from the affected limb [1,2]. Next, we analyzed the training effect during the four separate training sessions and the effect of stimulation on the subgroup with MEPs and the subgroup without MEPs (No-MEP). It should be considered that the No-MEP group consisted of N = 2 participants and the MEP group of N = 9 participants only. Therefore, this uneven separation in groups rendered the data unbalanced with in part very small, sampled subgroups. Thus, we have refrained from an inferential statistical analysis. The descriptive statistics suggest that the averages of the performance during the training sessions demonstrated a difference between MF-stimulation with respect to control stimulation in the no-MEP group, with a faciliatory effect of MF-stimulation on training. This was not as evident in the MEP group, please see also Fig. S4 below. This might indicate a higher sensitivity for CB-stimulation in participants with a no-MEP status.

**Fig. S4 Exploratory responder analysis based on the presence of MEPs (no vs. yes).** Subjects #02 and #08 had a no-MEP status. Training performance is shown as AUC corrected to baseline. More negative values indicate better performance. The tDCS effect seemed to be pronounced for stroke survivors with a no-MEP status. Error bars correspond to standard error of the mean (SEM).

# References

1. Barker AT, Jalinous R, Freeston IL. Non-invasive magnetic stimulation of human motor cortex. Lancet. 1985;1:1106–7.

2. Rapisarda G, Bastings E, de Noordhout AM, Pennisi G, Delwaide PJ. Can motor recovery in stroke patients be predicted by early transcranial magnetic stimulation? Stroke. 1996;27:2191–6.
